# Systems and policy factors affecting service delivery for homeless adults with intellectual and developmental disabilities

**DOI:** 10.3389/fpsyt.2026.1841192

**Published:** 2026-07-15

**Authors:** Whitney Thurman, Elizabeth Heitkemper, Tara Hutson

**Affiliations:** 1 School of Nursing, The University of Texas at Austin, Austin, TX, United States; 2 School of Nursing, Biobehavioral Nursing & Health Informatics, University of Washington, Seattle, WA, United States

**Keywords:** care coordination, homelessness, public policy, system churn, systems

## Abstract

**Background:**

Intellectual and developmental disability (IDD) is characterized by impairment of cognitive function that results in daily living limitations. Adults with IDD face barriers to meeting basic needs and must navigate multiple public systems to access services. The housing affordability crisis has contributed to a disproportionate overrepresentation of adults with IDD who experience homelessness. Little evidence has articulated the unique systems– and policy–level issues facing homeless adults with IDD.

**Objective:**

The purpose of this study was to 1) map the systems with which adults with IDD who experience homelessness must interface in order to access needed services and 2) describe the systems- and policy-level issues impacting service delivery and care coordination of this population.

**Methods:**

This qualitative study interviewed professionals providing disability, homeless, and/or social care services to adults experiencing homelessness between March and June 2021. Data were analyzed using qualitative content analysis.

**Results:**

Participants (*n* = 18) mostly identified as female (*n* = 11), the mean age was 44 (13.9), and included clinicians (*n* = 5), case managers (*n* = 7), allied professionals (*n* = 5), and outreach specialists (*n* = 1). Participants identified five unique systems with which individuals at the intersection of IDD and homelessness frequently interact to access care and services. The theme structurally fragmented care for complex needs captures participants’ perceptions of systems-level issues, and the theme policy-driven gaps in care encompasses perceptions of policy-level issues.

**Conclusion:**

Homeless adults with IDD exist in a nearly perpetual process of cycling through public systems, and health equity cannot be attained without attention to the structural ableism that is embedded throughout. Findings highlight the importance of moving away from a crisis–driven model of care to a cohesive care ecosystem, and future research is needed to develop and test models of care that eliminate system churn so that all individuals are supported.

## Introduction

Homelessness in the United States (U.S.) affects an estimated 2.5–3.5 million individuals annually, and over half of adults experiencing homelessness live with at least one disability (1). Within this broader population, people with intellectual and developmental disabilities (IDD) represent a particularly vulnerable and underserved group. IDDs are characterized by life-long impairment in cognitive function in language, learning, mobility, self-direction, capacity for independent living, economic self-sufficiency, or self-care (2). Despite the fact that many people with IDD live healthy, meaningful lives, evidence indicates that this group is vastly overrepresented in homeless populations. While exact prevalence remains unknown, estimates range from 12% to 39% (3), and prior research has identified rates of 20% among a sample of 90 adults experiencing homelessness in the U.S. (4) and 16% among a sample of 567 youth experiencing homelessness in Canada (5). Despite the scale of this overlap, homelessness among people with IDD remains a critically understudied issue.

Pathways into homelessness among people with IDD are complex and shaped by intersecting systems of disadvantage operating across individual, social, economic, and political domains. An intersectional lens underscores how disability interacts with other marginal structural positions to compound vulnerability. For example, in the U.S., one of the most significant drivers of homelessness is the ongoing housing affordability crisis characterized by rising rents, inadequate supplies of deeply affordable housing, and insufficient rental assistance for extremely low-income households (6). For people with IDD, these structural pressures are intensified by economic marginalization and inequitable labor market participation. In the U.S., 17% of adults with IDD report employment in a paid community job compared to the 62.5% labor force participation rate among the general population (7). Consequently, many adults with IDD rely on publicly funded income such as Supplemental Security Income (SSI) to meet living expenses. However, SSI provides an average monthly benefit of $743, an amount that is insufficient to secure stable housing in any U.S. market without additional subsidies (8). Structural inequities in access to housing assistance further exacerbate risk. Although millions of disabled adults qualify for housing supports, many do not receive them due to limited program capacity and systemic access barriers (9), leaving them at elevated risk for homelessness or institutionalization.

Beyond income, people with IDD face additional vulnerabilities that increase their risk of losing stable housing. Functional limitations in areas such as communication, executive functioning, and self-advocacy can make navigating complex service systems, understanding housing contracts, or maintaining tenancy requirements difficult without ongoing support (3). Family-based caregiving, the primary form of caregiving in the U.S., represents another point of vulnerability. Prior research has identified the caregiving burden and stigma associated with IDD as contributing to family instability (10, 11) and that adults with IDD often lose their housing when their parents or other family caregivers are no longer able to care for them (12, 13). These moments of vulnerability are compounded by co-occurring serious mental illness, substance use disorders, chronic health conditions, and trauma, all of which characterize the high-acuity needs of people at the intersection of IDD and homelessness (14).

In this context, the ability to secure and sustain housing is not solely a function of individual capacity but is deeply shaped by the availability and coordination of formal support systems. Many adults with IDD require lifelong supports to meet basic needs and to maintain stable housing in the community. Accessing such supports typically requires navigating multiple public systems such as disability services, healthcare, and housing assistance which are fragmented and difficult to access even for individuals without cognitive or adaptive impairments (15). Thus, the health and social services workforce plays a central role in supporting people with IDD. Case managers, social workers, clinicians, and direct support professionals serve as primary conduits between individuals with IDD and the complex array of publicly funded services on which they depend. Case management is a lynchpin in the system of supports for people with IDD as it is essential that someone is responsible for coordinating information exchange and interdisciplinary collaboration (16, 17). For people with IDD who are experiencing homelessness, this coordination function is especially critical as individuals at this intersection must navigate multiple systems that do not traditionally work together.

Despite the centrality of the workforce to service delivery, providers across these systems face significant barriers to serving this population well. Healthcare providers receive limited training and thus have limited knowledge of IDD (18). The situation is similar in homeless services: providers struggling to serve this population cite lack of training and awareness of specific needs, fragmented systems, and inadequately funded healthcare and housing supports as key barriers to appropriate and accessible service delivery (12). When cross-sector collaboration does not routinely occur, the responsibility often falls on individuals, their caregivers, and their family members to coordinate complex care. This task can be insurmountable for people with IDD without robust support networks.

Taken together, the evidence points to a significant systems-level challenge: people with IDD experiencing homelessness have complex, multi-domain needs that exceed the capacity of any single system. In the U.S., homelessness response systems are typically designed to address acute housing crises, while disability service systems focus on long-term supports for individuals who are stably housed. When individuals with IDD become homeless, they frequently fall between these systems, encountering service gaps, unclear responsibility, and fragmented care pathways (19, 20). Despite growing recognition of these issues, little empirical work has examined how professionals working across disability, homelessness, and social care systems understand and navigate the policies and structures that shape service delivery for homeless adults with IDD. Most existing studies focus on individual experiences or intervention outcomes, leaving a critical gap in understanding the systems–level dynamics that facilitate or hinder access to needed services and supports.

### Purpose

The purpose of this study was to 1) map the systems that participants identify as impacting access to care and services for adults with IDD who experience homelessness and 2) describe participants’ perspectives of the systems- and policy-level issues impacting service delivery and care coordination of this population. By centering the perspectives of professionals working at the intersection of disability, homelessness, and social care, this study aims to illuminate how policy design, funding priorities, and cross-sector relationships shape the provision of services and supports. Understanding these intersecting dynamics is essential for informing the development of integrated care models that move beyond crisis-driven responses toward coordinated, equitable systems of support that are responsive to the diverse needs and lived realities of people with IDD.

### Theoretical framework

To guide analysis and interpretation, we used the Social Ecological Model (SEM). The SEM conceptualizes health and social outcomes as the product of dynamic interactions across multiple, nested levels of influence including the individual, interpersonal, organizational, community, and policy or societal levels. Rather than locating responsibility for outcomes solely within individuals, the SEM emphasizes how broader structural, institutional, and policy environments shape opportunities, constraints, and lived experiences.

The SEM is particularly well suited to examining homelessness among people with IDD because homelessness is not solely an individual’s housing status but is instead a condition produced through the interaction of policies and structural conditions. In this analysis, the SEM was used to organize attention to systems- and policy-level influences while remaining attentive to how these forces shape service delivery and care coordination at organizational and interpersonal levels. By situating participant perspectives within a social ecological framework, this study moves beyond individual-level experiences and explanations to highlight the structural conditions and policy environments that shape service access and delivery.

## Materials and methods

Because little was known regarding the systems and policies that dictate services provided to people with IDD who experience homelessness, we used a qualitative descriptive design. This approach is well-suited to exploratory research such as the current study because it produces rich, low-inference descriptions grounded in participants’ experiences and perspectives (21, 22). The study was deemed exempt by the Institutional Review Board at the first author’s university (Study 00000755).

### Sample, setting, and recruitment

Individuals were eligible to participate if they were at least 18 years old and had been involved in the provision of homeless services or disability services for at least 12 months. Participants were recruited from Texas, United States. Several methods were used to recruit participants including sending informational emails through a community listserv used by homeless services providers in the central Texas region, directing emails to professional contacts, and by word of mouth. Potential participants who were interested in learning more were directed to a Qualtrics survey that included the study’s purpose, details, expectations, and an option to assent to participation. Upon assent, the survey proceeded with demographic questions and scheduling of interviews. For those who did not assent, the survey ended by thanking the individual for their time and consideration. Interviews were conducted between March and June 2021, and each participant received a $40 gift card in recognition of their time and expertise.

### Data collection

Each interview was conducted remotely using Zoom by the first or second author and lasted approximately 60 minutes. Audio recordings were professionally transcribed and verified by a graduate student prior to analysis.

### Data analysis

Data were analyzed using qualitative content analysis that integrated inductive and deductive coding approaches. NVivo qualitative data management software was used to facilitate data organization and analysis. Analysis began with an inductive approach to remain grounded in participants’ accounts and to allow patterns to emerge from the data without imposing *a priori* categories. The research team first jointly coded one transcript to develop preliminary codes, discussing interpretations, meanings, and boundaries. Each team member then independently coded a second transcript using the initial codes while adding new codes as needed. The research team met regularly to compare coding, resolve discrepancies, refine code definitions, and develop a coherent coding framework. This framework was applied across transcripts, with continued openness to refinement as analysis deepened. As analysis progressed, deductive coding was incorporated to examine how emergent patterns aligned with broader conceptual domains informed by the SEM, particularly those related to organizational practices, inter-system relationships, and policy contexts. For the final 25% of coding, inductive and deductive processes were applied iteratively rather than sequentially, allowing data-driven insights to inform conceptual categorization while remaining attentive to participants’ language and meanings. This resulted in the content of the system map, themes, and categories coming from a purely inductive coding approach, whereas the identification of the relationships between the map systems, map core elements, themes, and categories was guided by a deductive coding approach using the SEM. Upon completion of coding, the team reviewed all codes and categories to assess meaning saturation and to examine relationships across systems- and policy-level domains (23).

#### Rigor, validity, and reliability

We incorporated several procedures for credibility, transferability, dependability, and confirmability throughout the research process in order to promote trustworthiness. These procedures included regular team debriefing, reflexive journaling, theoretical memoing, consideration of negative cases, and maintenance of an audit trail using NVivo. The research team also engaged in ongoing reflexive discussion regarding professional roles, perspectives, and assumptions related to disability, homelessness, and service systems.

## Results

Eighteen individuals were interviewed (**Table 1**), 61.1% of whom were female with a mean (SD) age of 44 (13.9) years. Nine participants worked in homeless services, 8 worked in disability services, and 1 participant reported working in both sectors. On average, participants had worked with their current employer for 5.8 years.

**Table 1:** General characteristics of participants (*N* = 18).

Participant characteristics	
Age in years, *M* (SD)	44 (13.9)
Gender, *n* (%)	
Female	11 (61.1)
Male	7 (38.9)
Type of Practice, *n* (%)	
Homeless Services	9 (50)
Disability Services	8 (44.4)
Both	1 (5.6)
Profession, *n* (%)	
Case Manager	7 (38.9)
Outreach Specialist	1 (5.6)
Allied Professional	5 (27.7)
Attorney	2 (11.1)
Administrative	2 (11.1)
Behaviorist	1 (5.6)
Clinician	5 (27.7)
Psychiatric Mental Health Nurse Practitioner	2 (11.1)
Registered Nurse	1 (5.6)
Physician	1 (5.6)
Licensed Psychologist	1 (5.6)

### Qualitative findings

#### Exosystem map as identified by participants

**Figure 1:**
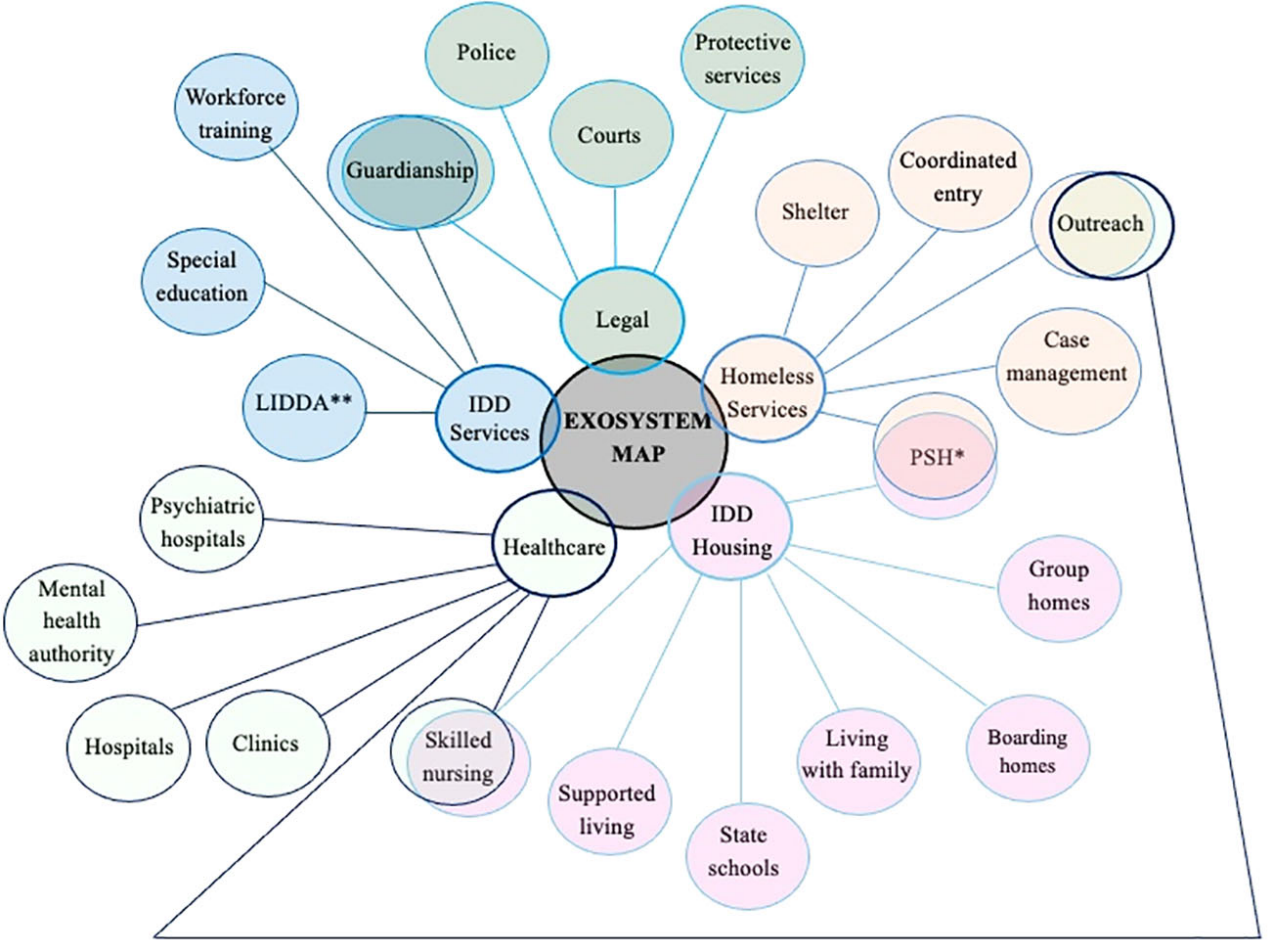
System map for adults at the intersection of intellectual and developmental disability (IDD) and homelessness according to participants. *Permanent Supportive Housing (PSH), **Local Intellectual and Developmental Disability Authority (LIDDA).

Participants identified five unique systems with which individuals at the intersection of IDD and homelessness regularly interact. They were: 1) homeless services, 2) housing, 3) healthcare, 4) IDD services, and 5) the criminal legal system (**Figure 1**). Through talking about their longstanding work with this specific population, participants described the range of core elements (e.g., organizations, policies, processes, programs, etc.) that they routinely encountered and worked with that were mostly unique to each system. **Figure 1** depicts each of these systems and their related participants identified as critical to their work with more detail below.

##### System 1: IDD Services

Participants identified four core elements as central to their work: *workforce training, special education, guardianship*, and the *Local Intellectual and Developmental Disability Authority (LIDDA)*. Workforce training was described as encompassing a range of programming, from short-term skill-building workshops to long-term vocational training. Special education referred to services provided under the Individuals with Disabilities Education Act (IDEA), a federal law enacted in the U.S. in 1975. Guardianship was discussed as spanning both the IDD and legal systems, as it involves a court-appointed decision-maker for individuals deemed incapacitated. Participants focused much of their discussion on guardianship, describing its role in clients’ lives as well as the vulnerabilities and challenges clients may experience because of guardianship arrangements.

##### System 2: Homeless Services

Participants identified five core elements as central to the homelessness response system and critical to their work: outreach, coordinated entry, case management, shelter, and permanent supportive housing (PSH). Outreach was described as essential for engaging people in unsheltered settings, building trust, addressing immediate needs, and connecting individuals to services. Coordinated entry was discussed as a standardized process used to assess, prioritize, and refer individuals to housing and services, with participants noting both its benefits and implementation challenges. Case management was emphasized by all participants as critical for helping clients navigate systems, access services, and work toward stable housing and self-sufficiency. Shelter was identified as an important option when other housing was unavailable, and PSH was described as a cornerstone of homelessness response, albeit one in short supply, combing non-time-limited affordable housing with supportive services to promote long-term housing stability.

##### System 3: IDD housing

Participants described seven housing options relevant to adults with IDD: PSH, group homes, boarding homes, living with family, state schools, supported living, and skilled nursing. PSH was discussed as an important but scarce option, with participants emphasizing the need for models specifically designed to meet the needs of adults with IDD. Group homes were described as common housing solutions, though participants noted wide variation in setting, services, and quality. Boarding homes were also identified as frequently used but were described as unlicensed and unregulated, often resulting in lower-quality living conditions. All participants emphasized the critical role of family, noting that clients who can live with family often experience better outcomes. State schools, also referred to as state supported living centers, were described as an option for a limited number of individuals requiring 24-hour care. Supported living was viewed as a preferred community-based option due to its individualized supports, though participants identified cost and limited availability as major barriers. Skilled nursing was described as an important housing option for clients with significant physical disabilities or complex medical needs acquired over the lifespan.

##### System 4: Healthcare

Participants identified six core elements within the healthcare system: skilled nursing, hospitals, outreach, the local mental health authority (LMHA), and psychiatric hospitals. Skilled nursing was described as a key point of engagement for clients with complex medical needs requiring 24-hour care. Clinics were reported as difficult to access, whereas hospitals and outreach were identified as more common sources of care. Participants noted that clients frequently relied on emergency departments or medical outreach services that provided care in unsheltered locations. The LMHA was described as critical for accessing public mental health services and was referred to as a gatekeeper for clients’ mental health care. Participants also described numerous challenges related to LMHA access and service delivery. Psychiatric hospitals were discussed as playing an important role in client stabilization, though participants highlighted significant barriers to access and concerns about the adequacy of care.

##### System 5: Legal

Participants described four core elements within the legal system that shaped their clients’ experiences: guardianship, police, courts, and protective services. Guardianship was discussed as both a supportive and restrictive mechanism. Participants noted that while guardianship could sometimes provide stability and safety, it limited client autonomy. Further, many adults experiencing homelessness do not have close, supportive relationships with family members who might otherwise assume guardianship. Police were identified as frequent points of contact for clients, and many participants described situations in which clients were repeatedly arrested and jailed for low-level offenses because they did not understand the consequences of some of their actions. Courts were discussed in relation to criminal charges, mental health diversion, and guardianship cases. Participants emphasized that court processes were often confusing and inaccessible for clients, particularly those without legal representation or adequate support. Protective services were described as an important system for addressing abuse, neglect, and exploitation. However, participants also highlighted challenges related to eligibility thresholds, response times, and limited follow-up. Overall, participants characterized the legal system as a critical but fragmented system that often intersects with other service systems and significantly influences client stability and housing outcomes.

#### Participants’ perceptions of systems-level issues

The theme *structurally fragmented care for complex needs* captures participants’ perceptions of the systems-level issues that impact service delivery and care coordination for their clients at the intersection of IDD and homelessness. Participants described a fragmented service environment that is poorly aligned with the complex, overlapping needs of individuals in this population. Rather than functioning as an integrated continuum, disability, healthcare, and homeless services operate as separate systems. Participants described rules that required individuals to navigate these systems sequentially, with access to one service contingent on engagement with another. This fragmentation placed substantial demands on participants and their clients and contributed to delays, unmet needs, and discontinuities in care. Three categories illustrate how structural features of the service landscape impeded care delivery and coordination: services require individual engagement, fragmented systems of care, and service mismatch at the intersection of systems.

##### Services require individual engagement

Access to services was contingent on an individual’s ability to actively engage with complex bureaucratic systems. Participants described how the systems within which they worked assumed a level of stability, motivation, capacity, or family support that many people with IDD who experience homelessness may not have. Expectations such as attending scheduled appointments, completing documentation, and navigating multiple referral pathways created substantial barriers to care. In describing her work with one client, a participant said,

“This client couldn’t remember appointments, so she wasn’t going to be successful at any agency that required you to show up at a certain time, on a certain day of the week, to see your case manager, which is 99% of all case manager organizations”.

A different participant shared her experiences with the local mental health authority,

“You can’t just walk into a clinic and say ‘I need help…’ You have to do an intake first. And it’s like twelve steps. And you have to go to a certain location for that. And then once you do your intake … there’s a couple more weeks before you’ll see a provider and then they’re just kind of like, try a medication”.

Participants noted that rather than accommodating cognitive impairment, housing instability, or competing survival needs, systems frequently required individuals to demonstrate consistent engagement before services could be accessed. One participant’s experience in trying to access services for a client at a community provider clearly demonstrated this phenomenon,

“you have to go through so many hurdles … it is first to be an established patient then to be referred to get an evaluation. You get that evaluation referral and then you meet with another person that just decides whether or not you’re appropriate to even then recommend for the evaluation”.

##### Fragmented systems of care

Participants consistently described a lack of coordination across homelessness, healthcare, disability, and social service systems resulting in fragmented and inefficient service delivery. Communication between agencies was constrained by incompatible documentation systems, restricted data sharing, and complex release-of-information (ROI) procedures. One participant who worked in homeless services described distinct processes for managing ROIs for three different organizations, and within one of those organizations she described two different processes,

“I probably handle 30 ROIs for [provider] every week. They have a verbal ROI if you just need to talk to somebody about scheduling. And then they have an actual ROI for, like, release of information. So, the verbal one is, I think, inclusive of scheduling appointments and talking to the doctor. Whereas the ROI is for medical records and what we call face sheets which is a big thing we do to help people get their social security cards”.

A different participant who worked in disability services described similar byzantine procedures for sharing information. When asked about how information sharing could be improved so that she could better support her clients, she said,

“we still have empty beds in our group homes. So if we have data … of people who are still on the street, we could reach out to them … and let them know there’s a place for you here”.

In the absence of formal integration—or as workarounds to unclear communication processes—participants described relying on informal networks and personal relationships to obtain needed information. These efforts were time intensive, inconsistent, and depended on individual knowledge and experience. One participant gave an example of calling upon a specific colleague at one community organization:

“Hey, can you look this client up real quick? Here’s the ROI, I just emailed it to you. I’m trying to verify their homelessness so I can complete their housing application”.

She went on to note that because of her ability to make that quick phone call, that client’s housing application was complete but without that call she said,

“I don’t know how in the hell I would have gotten that info”.

A different participant who worked in outpatient healthcare detailed her approach to when she needed more detailed social histories for specific patients:

“Oftentimes when I get stuck, I’ll go to the hospital, and I’ll read social work notes. Because there will be nuggets of great information … even just knowing that this guy came from [different state] and might have had benefits there”.

##### Service mismatch at the intersection of systems

A pervasive challenge identified by participants was the lack of services designed for individuals whose needs span multiple systems, resulting in a mismatch between available programs and client needs. Most services were structured to address a single domain such as mental health, homelessness, or disability without accounting for the co-occurrence of IDD, chronic health conditions, and housing instability. Notably, participants perceived that even the entities responsible for IDD services had very little to offer individuals experiencing homelessness. One participant said,

“They [local mental health authority] did make the formal diagnosis of IDD which was helpful for her disability application, but past that, they really had nothing to offer. I think they try to offer some supportive services for people who are living with their families, but they don’t really have anything for people experiencing homelessness. And there’s a pretty shocking amount of people with IDD experiencing homelessness outside”.

As a result of this service mismatch, individuals were often placed in programs that were not aligned with their needs. One participant described ongoing housing challenges with clients that have dual diagnoses of IDD and mental illness. He said,

“I’ve had more than one client voluntarily agree to stay in the hospital and ask to stay in the hospital because there’s really very limited options on where to send them”.

Participants uniformly lamented the lack of housing resources designed for this population noting that many of their clients required a level of support that is not typically available and without which they are at extreme risk of exploitation. Multiple participants described unlicensed board and care homes as one of the only housing options available for their clients at this intersection of IDD and housing instability before going on to describe the dire conditions found there. One stated,

“I don’t know if you have any experience seeing what those are like in Texas, but they are just absolutely deplorable … people walking in soiled diapers with cigarettes all over the floor and everything … this is a housing reality that a lot of folks with IDD face”.

#### Participants’ perceptions of policy-level issues

The theme *policy-driven gaps in care* captures participants’ perceptions of policy-level issues. Participants described a policy environment in which the underlying logic of regulation and funding is misaligned with the needs of individuals at the intersection of IDD and homelessness. Rather than facilitating access to appropriate and sustained care, policies often introduced barriers, prioritized short-term crisis response, and allocated resources based on funding structures rather than population need. This results in a system where services are organized around administrative and fiscal priorities, rather than the complexity and continuity of care required by the population. Three categories illustrate how these dynamics operate in practice: *current policies harm more than help, policies focus only on acute problems*, and *funding priorities, not need, dictate services.*

##### Current policies harm more than help

This category reflects participants’ perceptions of policy environments that, rather than facilitating access to care, produce unintended barriers and exclusions through eligibility criteria, documentation requirements, and benefit structures. Across interviews, participants recounted repeated, unsuccessful attempts to connect clients to needed services due to policy constraints. These barriers were not described as isolated challenges but as routine features of the system that limited access even when needs were clearly identified. As one participant noted,

“I would love to connect people to appropriate services, but there’s no way”.

He went on to specify that the barriers stemmed from

“how people qualify for IDD [services]”.

Participants also described the consequences of the stringent eligibility criteria required to access disability-related supports. They described how formal diagnoses, historical records, and standardized assessments were necessary to qualify for services, yet these requirements were particularly difficult to meet for individuals experiencing homelessness. One participant succinctly stated,

“I think the biggest barrier has been attaining school records or past diagnoses of IDD”.

A different participant expanded on this saying,

“the barriers to getting somebody into IDD services … whether they’re older and they don’t have the school records and they’re so alienated from any type of family or people that they’ve known over the years that nobody can be that verification”.

These requirements frequently delayed or prevented access to needed supports altogether. In some cases, individuals with clear functional impairments were unable to qualify for services because they did not meet narrowly defined criteria or could not produce the required documentation. One participant shared this experience,

“so in her records from jail and other places she had a diagnosis of borderline intellectual functioning all over her medical records. But because nobody was going to order the school records—like you’re in jail, they don’t care that much—then they can’t say she has IDD … but that official diagnosis, if you’re trying to get benefits, is very important”.

##### Policies focus only on acute problems

This category encompasses participants’ perceptions of their policy environments as primarily oriented toward crisis response rather than prevention or long-term support. Programs and funding streams were designed to address immediate, high-acuity needs, often resulting in institutional responses such as hospitalization or incarceration. Consequently, individuals typically accessed services at points of crisis, with limited access to ongoing, community-based supports. Even when services were secured, participants noted that they often did not align with individuals’ needs or preferences. As one provider reflected, many clients expressed,

“This is not what I wanted. This is not what I chose for myself”,

yet

“they have to get in trouble to get the help”.

This crisis-oriented structure contributed to recurrent cycling across systems, with individuals moving between emergency departments, psychiatric facilities, shelters, and the criminal justice system without achieving stability. One participant described this cycling,

“so now we have people that are constantly getting picked up and sent to inpatient psychiatric treatment because they can’t properly take their medications. Either there’s no one there to help them or they refuse and there’s no one there saying, ‘let’s take this everyday.’”

Others described how the recurrent cycling through systems itself undermined stability due to inconsistent care and the lack of coordinated care across facilities. For example,

“there was another individual that had gone to a behavioral hospital … and they took him off all of his medications. Every single medication that he had, completely taken off”.

Participants also emphasized that the absence of long-term support options frequently prolonged system involvement. In some cases, individuals remained in jail despite eligibility for release because no appropriate housing or support arrangements were available. As one participant explained,

“the prosecution is willing to dismiss the charges, but they have nowhere to go … can’t go back home and don’t have any other system of support”.

##### Funding priorities, not need, dictate services

Participants highlighted inconsistencies in access driven by funding structures, where eligibility and service availability varied substantially depending on the source of funding. One participant described how two individuals with the same diagnosis could have vastly different service trajectories: one might receive immediate placement in a supportive group home, while another could face a 10- to 15-year waitlist for comparable services. These disparities were perceived as arbitrary and disconnected from actual need. A different participant detailed how funding structures created access barriers when she described the difficulty accessing services from the local MHMR:

“I think it’s not their fault. It’s how their funding is structured that they have to put all these things in place. So, it’s really tough … to get funding you’ve got to check all these boxes”.

## Discussion

This study provides a systems-level examination of how public service structures shape access to care for adults with IDD who experience homelessness. This study mapped the public systems that providers working across disability, homeless, health, and social service systems identified as relevant and examined their perceptions of the systems- and policy-level issues that shape service delivery and care coordination for homeless adults with IDD. While the map of the exosystem is hyperlocal and not necessarily inclusive of all public systems nor reflective of all jurisdictions, it nevertheless represents compiled snapshots of each of our participant’s perspectives and is therefore helpful in demonstrating system fragmentation and provider blind spots. The identification of five overlapping systems—homeless services, housing, healthcare, IDD services, and the criminal legal system—reinforces the ecological complexity of service navigation. Consistent with prior research, findings demonstrate that individuals at this intersection encounter fragmented systems that are not designed to address co-occurring disability, housing instability, and health needs (19, 20). By centering provider perspectives, this study extends existing literature by elucidating how policy design, funding structures, and inter-system misalignment actively produce barriers to care, rather than merely failing to resolve them.

A central contribution of this study is the identification of system churn as a defining feature of service engagement for this population. Participants described how their clients repeatedly cycled through emergency departments, inpatient psychiatric facilities, jails, shelters, and other public systems, reflecting structural conditions rather than individual disengagement. Beyond its inefficiency and cost, this system churn disrupts continuity of care and results in the loss of critical supports, including Medicaid and Supplemental Nutrition Assistance Program benefits (24). These findings align with prior research demonstrating that high utilizers of public systems often have unmet, complex needs that are poorly addressed by episodic care models (25, 26). Although prior research has identified common characteristics among high utilizers of public systems, the prevalence and role of IDD within this population remain underexplored (27, 28). Our findings indicate that cognitive and adaptive limitations associated with IDD likely contribute to and intensify challenges related to care continuity, service navigation, and adherence to administrative requirements, thereby increasing vulnerability to repeated system involvement and homelessness. Thus, without explicit attention to IDD within high-utilizer populations, interventions aimed at reducing system churn risk overlooking a subgroup with distinct and persistent support needs.

Findings also illustrate that system churn is not solely the result of individual-level vulnerabilities but is structurally produced through misaligned policies and fragmented service delivery systems. In the U.S., disability, homelessness, and healthcare systems operate within distinct administrative and funding structures, and individuals with IDD who become homeless often fall into the gaps between them. IDD services, primarily funded through Medicaid Home and Community-Based Services (HCBS) waivers, are designed for individuals who are stably housed and connected to family or long-term caregivers (29). Homelessness systems, primarily funded through the U.S. Department of Housing and Urban Development’s (HUD) Continuum of Care (CoC) program, are designed to address housing crises and do not include IDD-specific services (30). This misalignment creates eligibility gaps that exclude individuals who do not fit neatly into either system, reinforcing patterns of instability and service discontinuity. HUD funding does not support group homes or other disability-specific residential models within CoC programs, while Medicaid waiver services are frequently inaccessible to individuals experiencing literal homelessness due to requirements related to documentation, consent, and care coordination (29). Similar barriers have been documented in studies examining access to long-term services and supports, where administrative complexity and restrictive eligibility criteria limit access for individuals with high needs (31). The present findings extend this work by demonstrating how these barriers are amplified in the context of homelessness.

Importantly, participants’ accounts reveal that prevailing models of care are fundamentally misaligned with the needs of individuals with IDD who experience homelessness. Requirements for consistent engagement, self-advocacy, and service navigation presume a level of functional capacity and social support that many individuals in this population do not possess. These findings are consistent with prior research highlighting the reliance on family caregivers and the vulnerability that arises when such supports are absent (10, 11). In the absence of adaptive service models, adults with IDD are effectively excluded from systems that require them to demonstrate the very capacities that their disabilities may limit. This reflects broader patterns of structural ableism wherein policies and programs are designed around assumptions of independence and competency, thereby marginalizing those who require ongoing support (32).

Consistent with national evidence on housing shortages and inadequate rental assistance (6, 9), housing was identified as a critical point of system failure. Participants described a lack of housing options that provide both affordability and appropriate levels of support. Existing housing models such as emergency shelters, permanent supportive housing, or group homes typically do not accommodate the combined needs of IDD and homelessness. Prior research has documented similar mismatches between service availability and need for individuals with co-occurring disabilities and behavioral health conditions (33). The current findings provide a more contextual understanding of how these mismatches are experienced in practice with individuals often being forced into inappropriate, unsafe, and unregulated care settings.

### Research implications

This study identifies several priorities for future research. First IDD must be made visible within homelessness and high-utilizer research. Although system churn and frequent use of emergency, healthcare, and criminal legal systems are well documented, IDD remains largely unexamined within these populations. Future studies should incorporate systematic assessment of cognitive and adaptive functioning to better understand how IDD shapes service trajectories, care continuity, and housing outcomes. Greater attention is also needed on the high prevalence of psychiatric comorbidities among individuals with IDD which are frequently overlooked due to diagnostic overshadowing and related assessment challenges (34). These co-occurring mental health conditions likely further increase vulnerability to homelessness particularly when they disrupt personal and family relationships that serve as critical sources of housing, social, and caregiving support.

Second, research is needed to develop and test integrated, cross-sector models of care that are explicitly designed for adults with IDD who experience homelessness. Interdisciplinary care management models, which have been identified as essential for individuals with complex needs may be particularly well suited to this population if adapted to address cognitive and functional limitations (35). Recommendations from the IDD Engaged, Aligned, and Leading (IIDEAL) project may be particularly relevant in this context (36). These community-identified recommendations include strategies for system redesign and policy reform to improve the health and well-being of people with IDD. Importantly, these recommendations identify co-location of health care, social services, housing support, and vocational services as a key strategy for reducing system fragmentation. The IIDEAL model also calls for enhanced reimbursement for all clinical encounters for patients with IDD and for investments in shared infrastructure to share data across service sectors (36). As this model gains traction in communities across the country, mixed methods and implementation studies should examine its effectiveness in reducing system churn and improving housing stability.

Third, future research should center the perspectives of people with lived experience of IDD and homelessness. Provider accounts illuminate structural barriers, but inclusive, participatory approaches are essential for understanding autonomy, decision-making, and service engagement from the standpoint of those most affected. Longitudinal research examining how policy environments and eligibility criteria shape access to housing and supports over time would further inform prevention and systems reform efforts.

### Policy Implications

Findings also point to several areas in need of urgent policy reform. For example, the Medicaid HCBS program structure is poorly aligned with the realities of homelessness. While recent policy guidance recognized housing stability as a health-related need and allows states to cover housing-related services such as tenancy supports and environmental modifications through HCBS waivers and other Medicaid authorities, these mechanisms remain fragmented, optional across states, and limited in scope because Medicaid cannot directly fund long-term housing costs (37, 38). Consequently, individuals with IDD who experience homelessness may qualify for disability-related supports but are nevertheless unable to access those services because they lack housing stability. Thus, our findings reinforce the need for disability-responsive housing policy.

Evidence-based models of supportive housing including Housing First offer promising directions but require intentional adaptation to address the specific needs of individuals with IDD (39). A potential policy solution is the creation of integrated housing-enabled HCBS models specifically designed for individuals with disabilities who experience homelessness. Such models would move beyond the traditional assumption that HCBS recipients are already stably housed by embedding housing stabilization directly into LTSS systems. States could braid Medicaid funding to provide housing-related services to support housing stability with HUD Continuum of Care funding, state housing trust funds, and supportive housing programs to address the non-reimbursable costs of housing itself. Aligning these funding streams could improve housing stability, reduce system fragmentation, and improve continuity of care (40, 41).

## Limitations

Several limitations of this study should be acknowledged. The sample was drawn from a single state in the United States which limits the generalizability of findings to jurisdictions with different IDD service structures, coordinated entry policies, or healthcare benefits. However, generalizability is not the goal of qualitative research. Instead, we have addressed transferability by providing thick description of the study context and the systems and policy environments in which participants worked. Although the study was conducted in a single U.S. state, detailed reporting of service structures, policy constraints, and cross-system interactions allows readers to assess the applicability of findings to other jurisdictions with similar service configurations. It may be seen as a limitation that we did not verify interpretations with the study participants, and it should also be noted that this study relied solely on provider perspectives. The experiences of homeless adults with IDD themselves were not captured. Future research should center the voices of people with lived experience of IDD and homelessness to ensure that system reform efforts are grounded in the perspectives of those most impacted.

## Conclusion

This study advances understanding of homelessness among adults with IDD by illuminating the systems- and policy-level forces that shape service access and care coordination. By centering provider perspectives across disability, homelessness, and healthcare systems, findings demonstrate that repeated cycling through public systems is not the result of individual failure but a predictable outcome of fragmented structures and misaligned policies. The findings underscore the limitations of crisis-oriented, siloed service models and highlight the need for coordinated long-term approaches that recognize the lifelong support needs associated with IDD. Structural reform that prioritizes continuity, accessibility, and equity are needed to support community-based living and optimal health and safety for individuals in this highly marginalized population.

## Data Availability

The raw data supporting the conclusions of this article will be made available by the authors, without undue reservation.
